# Solution-processed negative gauge factor PtSe_2_ strain sensors†

**DOI:** 10.1039/d5nr01217a

**Published:** 2025-07-08

**Authors:** Cansu Ilhan, Eoin Caffrey, Shixin Liu, Jose Munuera, Zdeněk Sofer, Iva Plutnarová, Michael A. Morris, Jonathan N. Coleman, Tian Carey

**Affiliations:** a School of Chemistry, CRANN & AMBER Research Centres, Trinity College Dublin Dublin 2 Ireland; b School of Physics, CRANN & AMBER Research Centres, Trinity College Dublin Dublin 2 Ireland careyti@tcd.ie colemaj@tcd.ie; c Instituto de Ciencia y Tecnología del Carbono INCAR-CSIC C/Francisco Pintado Fe 26 Oviedo 33011 Spain; d Department of Inorganic Chemistry, University of Chemistry and Technology Prague Technická 5 Prague 6 166 28 Czech Republic

## Abstract

We undertake electrochemical exfoliation of a 2D semiconductor platinum diselenide, PtSe_2_ and investigate the piezoresistance response of a solution-processed network. Due to the large PtSe_2_ aspect ratios, exceeding 300, we achieve conformal flake-to-flake junctions and good inter-flake electrical coupling. Our measured piezoresistive gauge factor is negative (−5.45), consistent with the intrinsic negative gauge factor of PtSe_2_. This negative network gauge factor implies that strain is transferred from the substrate to the nanosheets. However, detailed modelling shows that the strain transferred to the nanosheets is much smaller than the applied strain, showing that conformal junctions do not necessarily lead to good mechanical coupling between nanosheets. Our model implies that this gauge factor is consistent with a strain transfer efficiency of 8.5%. Our strain sensor also demonstrated a cyclic response for over 1000 cycles, enabling the sensor to be used in future flexible optoelectronics applications.

## Abstract 2

We exfoliate PtSe_2_ and study its piezoresistive response in a solution-processed network. High aspect ratio flakes (>300) yield conformal junctions and a negative gauge factor (−5.45) even after >1000 strain cycles, ideal for flexible electronics.

## Graphical abstract

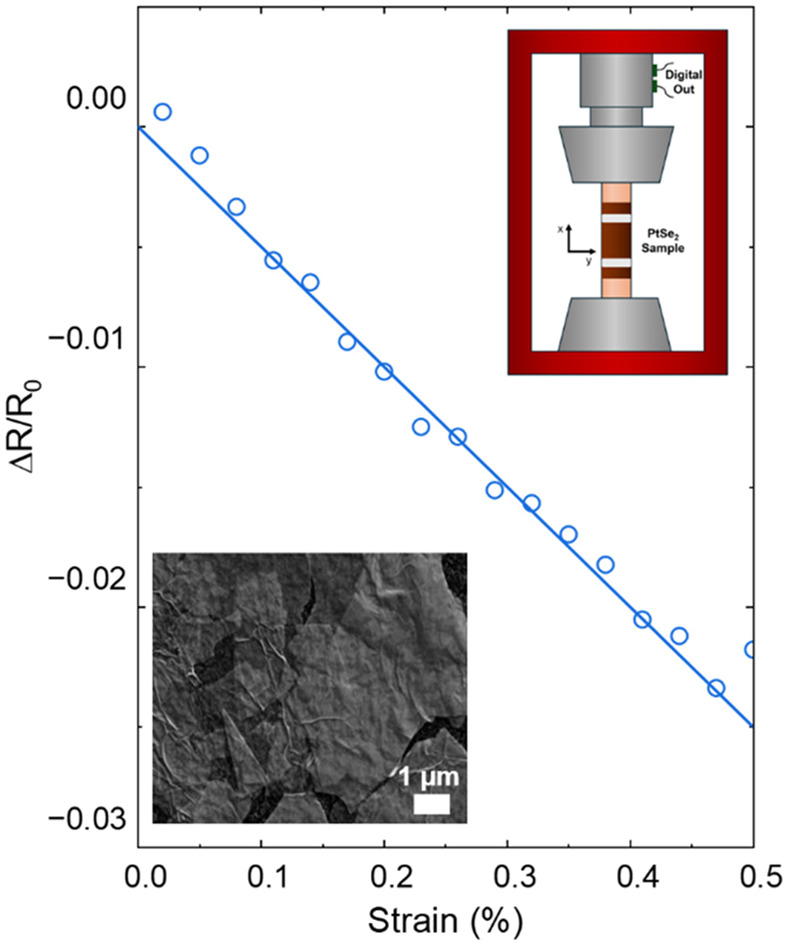

Piezoresistance is the property of materials which results in changes to their electrical resistance in response to applied mechanical strain. The size of the piezoresistive response is generally quantified by the gauge factor (*G*), which is defined as:^1^

1a Δ*R*/*R*_0_ = *Gε*

For an isotropic piezoresistive material, the gauge factor is given by^1^1b
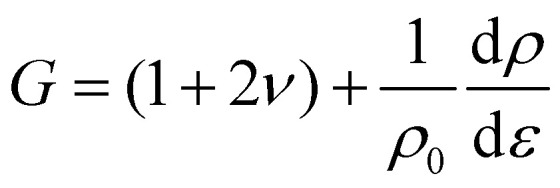
where *ν* and *ρ* are the Poisson ratio and resistivity of the piezoresistive material and *ρ*_0_ is its zero-strain resistivity. Strictly speaking, eqn (1a) and (1b) only apply in the limit of low strain. The term in brackets describes the effect of strain on resistance *via* dimensional changes. The second term describes the effect of strain on the intrinsic properties of the material itself. Because the first term is usually small, typically ∼2 (ref. 1), the magnitude of the gauge factor is often dominated by the second term, which can be much larger. For most crystalline materials d*ρ*/d*ε* is determined by the effect of strain on band structure and the resultant strain dependence of carrier density or mobility. For example, such effects lead to p-type silicon displaying a gauge factor of up to 175.^1^ It is less well known that the gauge factor can also be negative. Notably, nickel has a gauge factor of *G* = −12 due to strain-induced conductivity enhancement.^1^ Additionally, a small number of semiconductors display negative gauge factors. Most well-known, n-type silicon^1^ has been demonstrated with *G* = −135, while gauge factors as high as −285 have been reported for Si nanowires.^2^

Piezoresistive materials are widely used in strain gauges: sensors that electrically respond to mechanical deformation. While such sensors also require stability, linearity, and frequency independence, the gauge factor is the most studied parameter.^3^ Basic metallic strain gauges have gauge factors of ∼2,^4^ while silicon-based sensors offer much higher values (50–200),^5^ enabling small strain detection. However, silicon's stiffness and brittleness make it unsuitable for certain applications, especially wearable sensing.^6^

The flexibility and conformability required for wearable sensors can be achieved using two-dimensional (2D) materials, a broad family which includes materials such as graphene and molybdenum disulfide (MoS_2_).^7^ Piezoresistance has been reported in many 2D materials. For example, single graphene nanosheets have a well-defined intrinsic gauge factor in the range of *G* = 2–3.^8,9^ However, individual MoS_2_ sheets have reported gauge factors which vary over an enormous range from 760 (ref. 10) to −225 (ref. 11) including various values in between.^12–14^ This broad range partly occurs because of the dependence of gauge factor on nanosheet thickness^10,11^ as well as doping level^14^ and defect content.^15^

A cost-effective and scalable method for producing 2D-based, flexible piezoresistive films is by solution–deposition onto substrates *via* spraying, inkjet printing, or Langmuir-type processes.^16,17^ This approach is compatible with flexible substrates due to low processing temperatures (<120 °C)^18,19^ and can create nanosheet networks with gauge factors up to 350 for graphene-based systems.^20^ Network gauge factors vary with thickness,^20^ composition^21^ and morphology. For example, disordered WS_2_ and WSe_2_ networks show *G* ∼ 20,^21^ while aligned MoS_2_ networks have low *G* ∼ 3 due to nanosheet sliding.^22^ In both of these cases, the gauge factors were largely decoupled from the intrinsic gauge factors of the nanosheets comprising the networks. The piezoresistive properties of solution-deposited 2D networks remain largely unexplored, presenting an opportunity to study piezoresistance in complex systems with both order and disorder at different length scales.

PtSe_2_ is a 2D material with intriguing electrical and piezoresistive properties, reported to exhibit a negative gauge factor (*G* = −85) due to strain-induced density of states changes.^23^ PtSe_2_ nanosheets have been produced *via* liquid phase exfoliation (LPE)^24^ and by electrochemical exfoliation (EE).^25^ However, little is known about their solution-deposited networks, with only one study reporting using LPE nanosheets of PtSe_2_ to print networks (conductivities up to 700 S m^−1^).^26^ Given its strong intrinsic piezoresistive properties and solution processability, studying the piezoresistance of PtSe_2_ in networks is of great interest. Negative *G* materials are rare, with only two papers on printed nanomaterial networks showing this effect (MoS_2_/polymer,^27^*G* = −25; Ni/polymer,^28^*G* = −30). Demonstrating solution-processed PtSe_2_ films with negative *G* would broaden existing research while extending our understanding of nanosheet network piezoresistance.

Here we examine the piezoresistive properties of very thin networks of highly aligned, high aspect ratio nanosheets of PtSe_2_ produced by electrochemical exfoliation. We find that such networks have a negative gauge factor consistent with that previously observed for individual PtSe_2_ nanosheets. This study aims to understand the origins of the negative piezoelectric response in networks of aligned PtSe_2_ nanosheets. Developing such understanding will be essential for the development of future flexible electronic devices using nanosheet networks.

## Results and discussion

### PtSe_2_ ink production and characterisation

**Fig. 1:**
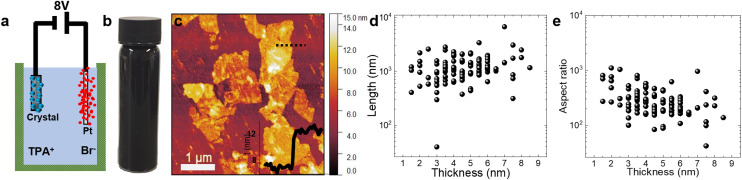
Electrochemical Exfoliation of PtSe_2_ and deposition by LS. (a) Schematic of EE process using a PtSe_2_ crystal and Pt foil as the electrodes. (b) Electrochemically exfoliated PtSe_2_ ink in IPA. (c) AFM image of the drop-cast PtSe_2_ flakes with a 4 nm apparent thickness. (d and e) Scatter-plot showing (d) individual flake lengths (mean = 1.3 μm) and (e) aspect ratios (mean = 312) plotted *versus* flake thickness.

Here, we used electrochemical exfoliation (EE) to produce PtSe_2_ nanosheets from bulk crystals. This method is particularly useful for producing nanosheets with relatively high aspect ratios.^29^Fig. 1a shows the schematic of the setup used in the EE step, where the crystal is located at the cathode, Pt foil was used as an anode, and −8 V was applied between the electrodes for 30 minutes to intercalate TPA^+^ cations into the PtSe_2_ crystal. During this process, the crystal expands to double its original volume,^30^ consistent with successful cation insertion. The expanded crystal was bath sonicated to disperse the expanded crystal in dimethylformamide (DMF) using polyvinylpyrrolidone (PVP) as a stabilizing polymer and then centrifuged to size select and wash the PtSe_2_ flakes. The resultant sediment was then redispersed in isopropyl alcohol (IPA) to make the PtSe_2_ ink shown in a bottle in Fig. 1b. Detailed protocols can be found in the ESI, Methods section.†

To assess the quality of the resultant PtSe_2_ flakes, atomic force microscopy (AFM) was used to characterise the dispersed phase after dropcasting on Si/SiO_2_ as shown in Fig. 1c. The AFM image shows a 4 nm thick flake and its cross-section. We measured the length and thickness of >100 nanosheets, as shown in Fig. 1d. The average measured flake thickness was 4.5 ± 1.6 nm, while the average flake length was 1.3 ± 0.8 μm. Additionally, the aspect ratio, defined as length/thickness, was calculated and plotted *versus* nanosheet thickness in Fig. 1e. This shows a broad range of aspect ratios between 100 and 1000, with an average aspect ratio of 300 ± 200. High aspect ratios are significant in the context of nanosheet networks.^31^ For nanosheets making up a network to conform to a rough surface, consisting either of a substrate below or other nanosheets within the network, one must consider the balance of adhesive energy (aligning the sheet to the surface locally) *versus* the bending energy (which resists the bending required to conformally map on to a rough surface). Previous calculations showed that, in order to conform to a rough surface without detachment, transition metal dichalcogenide nanosheets must have aspect ratios above about 40.^31^ This means that our relatively high aspect ratio nanosheets should be able to conform to their neighbours, yielding a highly aligned network.

**Fig. 2:**
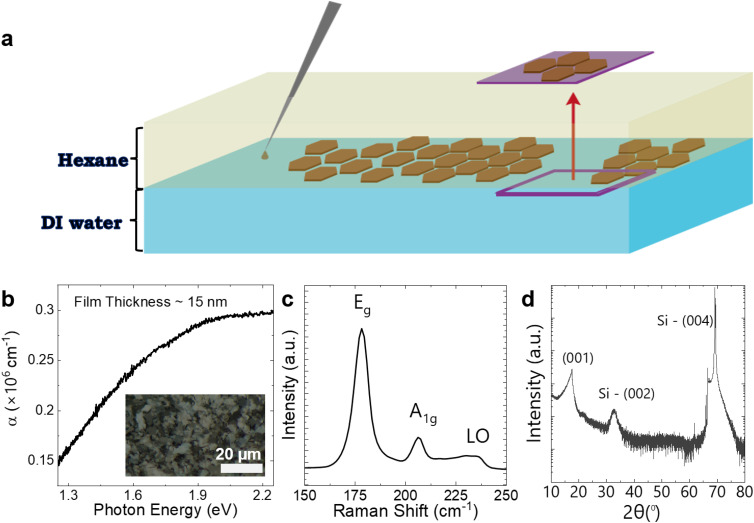
LS deposition and characterization of PtSe_2_ networks. (a) Schematic of a single LS deposition. The PtSe_2_ network is extruded from the water–hexane interface. (b) Absorption coefficient as a function photon energy for a 15 nm PtSe_2_ network deposited on PET after single LS deposition. Optical microscopy of PtSe_2_ network in the bright field shown in the inset, the scale bar is 20 μm. (c) Raman spectra of PtSe_2_, showing characteristic peaks. (d) XRD characterization of PtSe_2_ networks deposited on Si/SiO_2_ substrate after single LS deposition. The vertical axis is displayed as a log scale.

We utilised a deposition technique known to facilitate nanosheet alignment, Langmuir–Schaefer (LS) deposition, to produce nanosheet networks (see ESI, Methods†).^30,32^ As depicted in Fig. 2a, the LS technique leverages the interfacial tension between hexane and deionised water to form an organised network of flakes.^33^ A single LS deposition step was applied to create a highly aligned network (≈15 nm thick) of PtSe_2_ on PET substrates using a minimal amount of PtSe_2_ ink (the deposition required less than 120 μL with a concentration of 2.5 mg mL^−1^). The PtSe_2_ networks were then annealed at a low temperature of 120 °C in a glovebox to remove any excess solvent.

We used optical microscopy (Olympus DSX1000 digital microscope) in the bright field (Fig. 2b, inset) to confirm that the TMD flakes covered the entire PET substrate. Optical transmission spectra of the PtSe_2_ network on PET were measured using an integrating sphere to remove the effect of scattering. The absorption coefficient (*α*) as a function of photon energy (eV) is presented for a 15 nm thick PtSe_2_ network in Fig. 2b. The data shows an increase in *α* with increasing photon energy with no evidence of a band-edge down to 1.3 eV. This is as expected for our samples, which contain few-layer nanosheets of various thicknesses. It is well known that monolayer PtSe_2_ is a semiconductor with a bandgap of ∼1.2 eV. However, as nanosheet thickness increases, the bandgap falls rapidly, with nanosheets becoming semi-metallic above ∼4 layers.^34^

Raman spectroscopy is employed to assess the lattice vibrations of the PtSe_2_ flakes in our deposited network, as shown in Fig. 2c. This technique confirms the characteristic vibrational modes of the flakes while carefully controlling the laser power (<100 μW) to avoid any damage of the PtSe_2_ network. This approach reveals two prominent peaks at 178 cm^−1^ and 206 cm^−1^, corresponding to the E_g_ in-plane vibration and the A_1g_ out-of-plane vibration modes, respectively. A subtle peak near 232 cm^−1^ is also identified, attributed to a longitudinal optical (LO) mode. The results align with findings previously reported in the literature.^35–37^

X-ray Diffraction (XRD) analysis, illustrated in Fig. 2d, was utilized to assess the crystalline plane orientations of the PtSe_2_ network after LP deposition on the silicon/silicon oxide (Si/SiO_2_) substrate. A prominent peak at 17.5°, corresponding to an interlayer spacing of 0.51 nm,^38^ is assigned to the (001) reflection of PtSe_2_, indicating a preferential in-plane orientation of the flakes within the network.^39,40^ The absence of additional peaks suggests that the network primarily consists of flakes lying flat on the Si/SiO_2_ substrate with minimal contributions from other orientations.

### Sensor measurements of PtSe_2_ networks

Because a material's piezoresistance is closely linked to its electrical properties, we first measured the electrical resistivity of our PtSe_2_ networks to be *ρ*_Net_ = 0.83 ± 0.05 Ωm. We have recently shown that for very low-porosity networks of high-carrier density nanosheets^32^ as we have here, the resistivity can be written as:

2 *ρ*_Net_ ≈ 2*t*_NS_(*R*_NS_ + *R*_J_)

where *t*_NS_ is the nanosheet thickness and *R*_NS_ and *R*_J_ are the resistance of the individual nanosheets and the inter-nanosheet junctions, respectively. This equation was derived by modelling the network as a set of linear resistance chains in parallel. The length of each chain and the number of parallel chains comprising the network depends on the nanosheet dimensions. It is for this reason that the nanosheet lateral dimension (*i.e.* their length) doesn't appear: doubling the nanosheet length halves the number of nanosheets in a chain but also halves the number of parallel chains across the width of the network, such that the length dependence disappears.

Eqn (2) reflects that charge carriers passing through a network must cross an inter-nanosheet junction every time they pass through a nanosheet (to get to the next nanosheet). If the junction resistance is high relative to the nanosheets, the network resistivity will be much higher than that of its constituent nanosheets.^32^ Previous measurements on PtSe_2_ networks have implied that *R*_NS_ ≪ *R*_J_,^41^ meaning these networks are heavily junction-limited. This allows us to approximate eqn (2) as *ρ*_Net_ ≈ 2*t*_NS_*R*_J_. Then, using the measured value of *t*_NS_ = 4.5 ± 1.6 nm, we can estimate *R*_J_ ≈ 90 MΩ. This value is considerably larger than values of a few MΩ, previously reported for junction resistance in MoS_2_ networks.^32^ This high value may be specific to PtSe_2_. As mentioned above, we expect the thinnest nanosheets in our sample to be semiconducting while the thicker ones are semimetallic. This may lead to Schottky barriers at the interfaces between semiconducting/semimetallic nanosheets, leading to enhanced junction resistances.

**Fig. 3:**
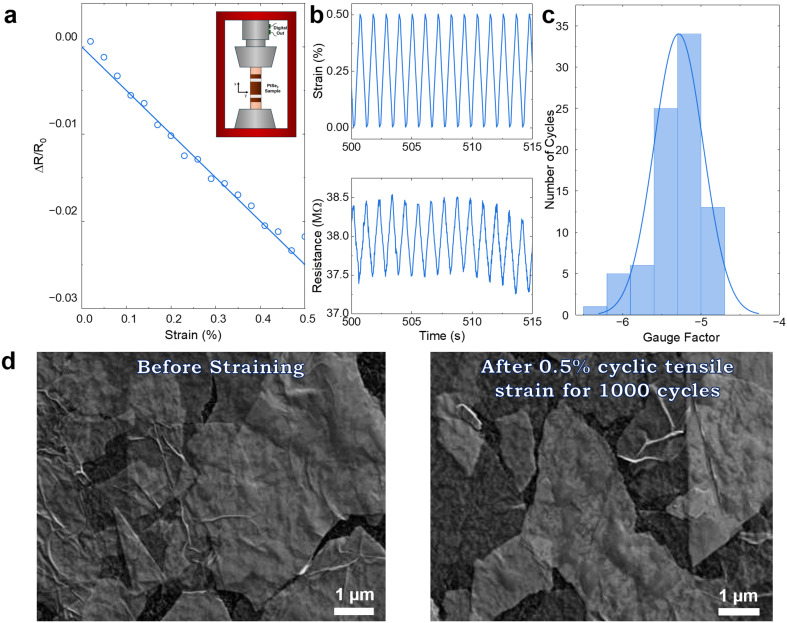
Sensor measurement of EE processes and single LS deposited PtSe_2_ network on PET. (a) Fractional resistance change plotted *versus* strain; the solid line is a linear fit. The inset shows a schematic of the tensile testing setup used in this experiment. (b) Cyclic resistance response of the network with 1 Hz. The sawtooth cycling profile can be seen in the top graph. (c) A histogram of a gauge factor of the PtSe_2_ network, the mean is −5.45 ± 0.33. Error found by standard deviation. (d) SEM verification of the PtSe_2_ flake distribution across the PET substrate after the single LS process both before the tensile strain was applied and after 1000 cycles of 0.5% cyclic tensile strain, consecutively.

We performed electrical measurements under tensile strain to assess the suitability of EE-processed and LS-deposited PtSe_2_ network for piezoresistive sensor measurements (ESI, Methods†). Before applying strain, we ensured the sample was taut at the beginning of the measurement process. The network exhibits a reduction in electrical resistance with applied tensile strain, as shown in Fig. 3a, indicating a negative gauge factor of approximately −5.0. This negative piezoresistive effect indicates a semiconductor strain gauge where the resistance can decrease as the material is stretched, which is contrary to the behaviour of standard metal foil strain gauges.^42^ The consistent linear behaviour up to 0.5% tensile strain highlights its potential for strain-sensing applications, particularly in the low-strain regime. Moreover, the sensitivity of the PtSe_2_ strain gauge, as reflected by the measured gauge factor (−5), was higher than that of some traditional metal foil strain gauges (2).^43^

Cyclic tests assessed the devices’ long-term stability, as shown in Fig. 3b. The graph in the upper panel of Fig. 3b depicts the strain profile of the cyclic electromechanical testing, with a triangular, sawtooth pattern from 0 to 0.5% strain and a strain rate of 1% s^−1^. The lower graph shows the corresponding changes in the resistance of the strain gauge over time. The periodic nature of the resistance changes correlates with the applied strain, confirming that the gauge is responding as expected to the applied strain. The histogram in Fig. 3c shows the distribution of calculated gauge factors from repeated measurements on an individual device. The histogram indicates that the gauge factors are normally distributed around a mean value of approximately −5.45 ± 0.33. The results are similar to those of Boland *et al.*, who also observed a negative gauge factor of up to −12 on films produced by converting a platinum metal layer to polycrystalline PtSe_2_ using a high temperature process.^44^ In addition, we note that Wagner *et al.* reported a negative gauge factor of −85 for PtSe_2_ films grown by thermally assisted conversion (TAC) and transferred onto polyimide foil. The negative gauge factor is attributed to an increase in the density of states in the PtSe_2_ under the application of strain.^23^

The LS deposition process ensures a highly aligned and densely packed flake structure, improving the strain transfer from the substrate into the PtSe_2_ network. Further verification of the PtSe_2_ flake distribution across the PET substrate after the single LS process was conducted using scanning electron microscopy (SEM) both before the tensile strain was applied and after 1000 cycles of 0.5% cyclic tensile strain (Fig. 3d). The PtSe_2_ flakes displayed a continuous and percolating network in each case, with flake-to-flake junctions that combine long overlaps and edge-to-edge contacts.

We note that the gauge factor measured in our solution-deposited networks, *G* = −5.45 is significantly lower than the values reported by Wagner *et al.* It is essential to understand the nature of this discrepancy. We attribute it to the networked nature of our films compared to the continuous films of Wagner *et al.* We can investigate this by developing a simple model to describe piezoresistance in our networks. This model is described in more detail in the ESI.†

We can combine eqn (1b), which is a general expression for piezoresistance, with eqn (2), which describes the resistivity of a network of nanosheets, to obtain the network gauge factor:3
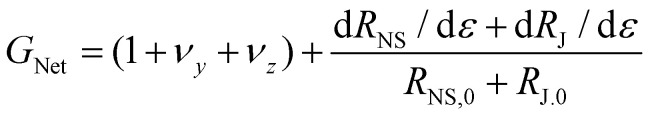


Here, the subscript “0” denotes the nanosheet and junction resistance values at *ε* = 0. In addition, we have modified the first term in eqn (1b) to take into account the fact that nanosheet networks are anisotropic and are expected to have different Poisson ratios in the in-plane (*y*) and out-of-plane (*z*) direction (here, the strain is applied in the *x*-direction).^22^

Eqn (3) is a general expression describing the gauge factor of a low-porosity network of highly aligned nanosheets, as we have here. This equation can be applied to various situations by considering the nature of the parameters d*R*_NS_/d*ε* and d*R*_J_/d*ε*. The simplest situation is that straining the network results in the nanosheets sliding past each other without becoming strained. Within this scenario, d*R*_NS_/d*ε* = 0, because the nanosheets themselves remain undeformed. This situation has been observed previously in aligned networks of electrochemically exfoliated MoS_2_ nanosheets.^22^ However, as shown in the ESI,† such a scenario can only yield a positive gauge factor within the network. This is because sliding reduces the junction area, thereby increasing junction resistance and, consequently, network resistance. Our experimentally measured negative gauge factor indicates that such a scenario cannot occur in these networks.

On the other hand, it is known from measurements by Wagner *et al.* on thermally grown PtSe_2_ films^23^ that the intrinsic gauge factor of PtSe_2_ is negative (*G*_NS_ = −85). Thus, our negative value *G*_Net_ implies that the nanosheets within our network are under strain. However, our experimentally measured network gauge factor (*G*_Net_ = −4.45) is much smaller than Wagner's value of *G*_NS_ = −85, implying that the strain in the nanosheets must be much smaller than the applied strain.

We can quantify these effects by developing a model for the gauge factor of nanosheet networks that incorporates a parameter describing the degree of strain transfer from the substrate, which is subject to the applied strain, to the nanosheets within the network. In doing this, we will not consider the mechanics of strain transfer, but will assume that strain has been transferred such that the (average) strain within the nanosheets (*ε*_NS_) is different to the applied strain (*ε*). Then we define a strain-transfer coefficient: *k* = *ε*_NS_/*ε* where we expect 0 ≤ *k* ≤ 1.

As shown in the ESI,† we begin by considering two overlapping nanosheets before and after the application of strain. The area of overlap is the inter-nanosheet junction. We presume that, after application of a strain to the substrate, the relative positions of the nanosheets are determined by *ε*, while their dimensions are determined by *ε*_NS_, and hence *k*, as well as *ν*_NS_, the in-plane Poisson ratio of the nanosheets. This allows us to calculate the area of the junction as a function of applied strain:4
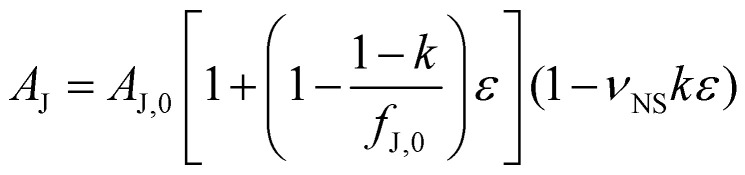
where *A*_J,0_ is the zero-strain junction area and *f*_J,0_ is the ratio of the junction area to the nanosheet area. We then assume that the junction resistance and area are inter-related by *R*_J_ = (*RA*)_J_/*A*_J_, where (*RA*)_J_ is a system parameter that describes inter-sheet charge transfer. The next step is to incorporate the expectation, supported by experimental data,^45^ that the inter-nanosheet charge transport is directly related to the nanosheet resistance: (*RA*)_J_ = *αR*_NS_, where *α* is an unknown parameter with units m^2^. This behaviour is expected as both inter-nanosheet and intra-nanosheet charge transport are controlled by the band structure, and both will be affected by straining the nanosheets. This allows us to link the junction resistance to the nanosheet resistance:5
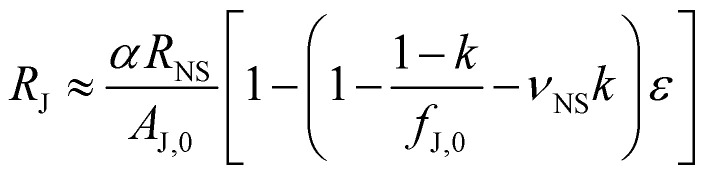


This equation can then be differentiated and then inserted into eqn (3). The resultant equation can be manipulated and simplified in various ways as described in the ESI,† to yield an equation for the network gauge factor as a function of various parameters, including *k*.6
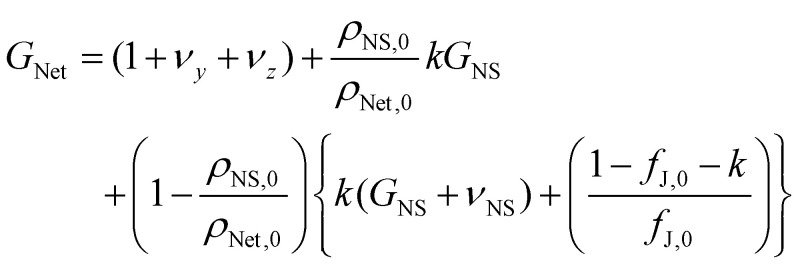


Here *ρ*_NS,0_ and *ρ*_Net,0_ are the unstrained resistivities of the individual nanosheets and the network, respectively. As shown in the ESI,† all parameters in this equation except *k* are known from the literature or can be estimated. This allows one to plot a graph of *G*_Net_*versus k* (Fig. S2†) which shows a linear progression for *G*_Net_ from ∼3 for the extreme case of no strain transfer (*k* = 0) to −83.5 for the other extreme of complete strain transfer (*k* = 1). Graphically, we can show that the experimental value of *G*_Net_ = −4.45 is consistent with a strain transfer of 8.5%.

We note that this situation differs from that observed previously in aligned networks of electrochemically exfoliated MoS_2_ nanosheets,^22^ where the data was consistent with perfect sliding of the nanosheets under strain and hence no strain transfer from the substrate to the nanosheet. We also emphasise that the value of 8.5% strain transfer is an average over the network. It is entirely possible that those nanosheets in contact with the substrate are strained to values much closer to the applied strain, but that the next layer of nanosheets experiences a lower strain, while the third layer feels an even lower strain, and so on. Likely, the mechanics of such a situation can indeed be modelled, using the procedure of Young *et al.* as a guide.^46^

## Conclusions

In this study, we successfully performed electrochemical exfoliation of the novel 2D semiconductor PtSe_2_ and explored the piezoresistance response of its solution-processed network. The high aspect ratios of PtSe_2_ nanosheets >300 enabled conformal flake-to-flake junctions, facilitating some strain transfer from the substrate to the network. This led to a negative gauge factor of −5.45. By developing a model for network gauge factor as a function of strain transfer efficiency, we can show that this gauge factor is consistent with a strain transfer efficiency of 8.5%.

## Author contributions

T. C. and J. C. conceived the idea. T. C. manufactured the inks and device. J. M. undertook AFM measurements. S. L. took all UV-vis measurements. E. C. conducted SEM imaging and carried out the piezoresistive testing. C. I. undertook XRD measurements and Raman Spectroscopy. All authors discussed the results. The manuscript was written by C. I., T. C., E. C. and J. C. in close consultation with other authors.

## Conflicts of interest

There are conflicts to declare.

## Supplementary Material

NR-017-D5NR01217A-s001

## Data Availability

The authors declare that the data supporting the findings of this study are available within the paper and its ESI files.† Data is also available from the corresponding author upon reasonable request.
