# Supplementary material for: Solution-processed negative gauge factor PtSe2 strain sensors
Source: Nanoscale. 2025 Jul 8;17(31):18083–91. doi: 10.1039/d5nr01217a (PMC12281280; doi:10.1039/d5nr01217a)
Supplement: NR-017-D5NR01217A-s001 [file NR-017-D5NR01217A-s001.pdf]

Supplementary Information (SI) for Nanoscale.
This journal is © The Royal Society of Chemistry 2025

Supporting Information

Solution-Processed Negative Gauge Factor $PtSe_2$ Strain Sensors

Cansu Ilhan[1], Eoin Caffrey[2], Shixin Liu[2], Jose Munuera[2,3], Zdeněk Sofer[4], Iva Plutnarová[4], Michael A. Morris[1], Jonathan N. Coleman[2], Tian Carey[2*]*

[1]*School of Chemistry, CRANN & AMBER Research Centres, Trinity College Dublin, Dublin 2, Ireland*

[2]*School of Physics, CRANN & AMBER Research Centres, Trinity College Dublin, Dublin 2, Ireland*

[3]*Department of Physics, Faculty of Sciences, University of Oviedo, C/ Leopoldo Calvo Sotelo, 18, 33007 Oviedo, Asturias, Spain.*

[4]*Department of Inorganic Chemistry, University of Chemistry and Technology Prague, Technická 5, Prague 6, 166 28, Czech Republic*

*Correspondence and requests for materials should be addressed to T.C. (careyti@tcd.ie)

Methods

Crystal Growth: $PtSe_2$ crystals were growth directly from melt in quartz ampoule. Pt (99.99%, sponge, Surepure, USA) and selenium (2-4mm granules, 99.9999%, Wuhan Xinrong New Technology Co., China) were placed in quartz ampoule (25x100mm, >3mm wall thickness) in stochiometric ratio together with 2 at.% excess of selenium and melt sealed under high vacuum (<$1x10^{-3}$ Pa using oil diffusion pump and liquid nitrogen trap). The ampoule was placed in muffle furnace and heated on 800 °C for 25 hours. Subsequently, the ampoule was heated on 1275°C within 4 hours and in 2 hours cooled on 1200 C and subsequently in 12 hours on room temperature. The ampoule were opened in argon filled glovebox and crystals mechanically separated. **WARNING:** During the reaction, significant pressure is formed in ampoule and thick walls are necessary to avoid pressure rapture of ampoule.

Electrochemical exfoliation of $PtSe_2$ crystals: The electrochemical exfoliation process started intercalating the $PtSe_2$ crystals. To incorporate $PtSe_2$ crystals, a two-electrode electrochemical setup is employed. The cathode comprises a small crystal piece measuring 5mm, while a platinum foil sourced from Alfa Aesar acts as the anode. Copper crocodile clips are used to secure the electrodes in the solution. The electrolyte solution consists of 5mg/mL tetra propylammonium (TPA^+) bromide obtained from Sigma-Aldrich mixed with 50 mL of propylene carbonate. Applying a voltage of 8V across the electrodes for 30 mins facilitates the intercalation of the 2D crystal with TPA+ cations. The observed expansion of the 2D crystal to over twice its original volume corroborates successful intercalation.

Ink formulation with 2D crystals: The expanded $PtSe_2$ crystal undergoes a series of treatments; it is first bath-sonicated (Fisherband 1122xx series) in a solution containing 1mg mL^{-1} PVP in DMF for 5 minutes. Then, centrifugation at 500 rpm (24g) for 20 minutes removes any unexfoliated crystals. The resulting dispersion is further size selected by centrifuging the supernatant (top 90%) at 1000 rpm (97g) for 1 hour and collecting the sediment. To eliminate the PVP, the sediment is diluted with 2mL of DMF and centrifuged at 10k rpm (9744g) for 1 hour, repeating this process twice and collecting the sediment each time. A third washing step, using IPA, removes residual DMF. The sediment is diluted in 0.5 mL of IPA and centrifuged at 10k rpm 9444g with the sediment collected afterward. The sediment is then redispersed in IPA (approximately 0.5 mL, concentration around 2.5 mg/mL) to create the 97g dispersion used for $PtSe_2$ in the study. IPA is chosen for its low boiling point (approximately 82.5°C), allowing for rapid evaporation after LS deposition.

$PtSe_2$ Network Formation by Langmuir-Schaefer: The LS assembly involves positioning Si/SiO_2 or PET chips (2 × 2 cm) on top of a Teflon stand (10 cm long), placed within a beaker containing approximately 100 mL of deionised water. A layer of distilled hexane (around 20 mL) is then deposited onto the water's surface to create a water/hexane interface, under which the chip is submerged. Next, inks of $PtSe_2$ are drop-cast (approximately 140 μL) onto the hexane surface until complete coverage is achieved. The Teflon stand and Si/SiO_2 chip are carefully manually extruded through the 2D crystal layer to coat the substrate surfacewith the TMD network. The resulting TMD networks are then allowed to dry in ambient air within a fume hood for approximately 6 hours. Manual extrusion during deposition and turbulent air flow in the fume hood during network drying helps to create flake-to-flake overlaps.

X-ray diffraction: XRD spectra were captured using a PanAlytical X'Pert Pro diffractometer with a Cu tube emitting Kα radiation (1.5406 Å). The spectra were acquired in the 2θ range from 10° to 80° on the networks prepared on Si/SiO_2 (100) orientated wafers (~300 nm oxide thickness).

Scanning Electron Microscopy: SEM imaging was conducted using a Carl Zeiss Ultra SEM. A secondary electron detector was used to obtain the images at a 3 kV accelerating voltage and 30 μm aperture.

Atomic Force Microscopy: AFM measurements were carried out with a Bruker Multimode 8 microscope to assess flake thickness and size. After diluting in IPA (1:100), $PtSe_2$ inks were dropcast on to Si/SiO_2 surfaces and then annealed at 120°C for 15 minutes in a N_2 glovebox. Employing OLTESPA R3 cantilevers in ScanAsyst mode allowed us to scan and statistically analyse x flakes, calculating their lateral size as the square root of length times width.

Raman Spectroscopy: Raman analysis was carried out using Witec Alpha 300R confocal Raman microscope with a laser wavelength of 532 nm at a power of < 300 μW and a spectral grating with 1800 lines/mm. The spectra shown for the sample were obtained by averaging 20 discrete point spectra.

Optical absorption spectroscopy: We acquired transmission spectra on LS networks using a Cary 50 UV-Vis spectrometer.

Electromechanical Testing: Mechanical testing was performed using a Zwick Z0.5 ProLine Tensile Tester fitted with a 100 N load cell. We attached silver wires to the $PtSe_2$ network,

which was deposited on PET through the LS method, using silver paint to form electrical connections. The dimensions of the samples were approximately 5 mm × 25 mm, and they featured a PET layer thickness of 130 μm. The strain sensor had a gauge length of 2 cm. Before testing, the sensors were subjected to a conditioning regimen that included cyclic stretching using a sawtooth strain profile. The devices were mounted into the clamps of the tensile tester, and the sensor was cyclically strained three times. This enabled the inter-clamp separation of zero strain to be identified (the point where the stress-strain graph showed an increase in stress with strain. The inter-clamp separation was set to this distance and the sensor was cycled a further three times to confirm that the electrical and mechanical responses were linear and functioning. Resistance measurements were conducted with a Keithley KE2601 Source-Meter, controlled through a 2-probe LabView configuration. The electromechanical testing was limited to a maximum strain of 0.5 % to avoid permanent damage such as cracking or electrode delamination.

Generating a model for gauge factor in networks of highly aligned nanosheets

The parameter which quantifies the magnitude of the piezoresistive effect is the gauge factor, G, which is defined as the fractional resistance change ($\Delta R/R_0$) per unit strain (ε) in the linear, low-strain regime, $\Delta R/R_0 = G\varepsilon$.[1, 2] Here, R_0 denotes the resistance in the unstrained state. Usually, the gauge factor is measured at low strain and in that limit it can be shown that for an isotropic material[3]

$$G = (1+2\nu) + \frac{1}{\rho_0}\frac{\mathrm{d}\rho}{\mathrm{d}\varepsilon} \tag{S1}$$

where ν and ρ are the Poisson ratio and resistivity of the piezoresistive material and ρ_0 is its zero-strain resistivity. The first term describes the effect of strain on sample dimensions (and so resistance) while the second term describes the effect of strain on the intrinsic material properties.

To properly analyse our data, it will be necessary to understand the factors controlling conduction in networks of highly aligned, electrochemically exfoliated nanosheets such as those under study here. Recently Gabbett et al proposed a model which well-describes conduction in all nanosheet networks.[4] This paper gives an equation for network resistivity which, in the case of very low-porosity networks of high-carrier density nanosheets [4] as we have here, can be written as (see SI of ref[4]):

$$\rho_{Net} \approx 2t_{NS}\left[R_{NS} + R_J\right] \tag{S2}$$

where t_{NS} is the mean nanosheet thickness and R_{NS} and R_J are the mean resistances of individual nanosheets and junctions within the network. Combining equation S1 and equation S2 allows us to generate an equation for the gauge factor of our networks. In addition to calculating the second term in equation S1, we modify the first term to take into account the fact that nanosheet networks are anisotropic and are expected to have different Poisson ratios in the in-plane (y) and out-of-plane (z) direction (here the strain is applied in the x direction):

$$G_{Net} = (1+\nu_y+\nu_z) + \frac{dR_{NS}/d\varepsilon + dR_J/d\varepsilon}{R_{NS,0} + R_{J.0}} \tag{S3}$$

Here the subscript, “0”, denotes the value at $\varepsilon = 0$. The first term describes the contribution to the piezoresistance from dimensional changes under deformation ($G_{Dimensional}$) while the second term describes the intrinsic piezoresistance of the material ($G_{Intrinsic}$). We stress that ν_y and ν_z apply to the network as a whole.

Now, we consider the second term in S3, focusing on networks such as those under study here which consist of highly aligned nanosheets with large area conformal junctions. We begin by calculating $dR_J / d\varepsilon$. To do this, we note that for aligned nanosheets with a well-defined junction area, the junction resistance is given by:

$$R_J \approx \frac{(RA)_J}{A_J} \qquad \text{(S4)}$$

Here A_J is the junction area that must depend on strain while $(RA)_J$ is a constant associated with transport across the junction. In figure S1, we model a junction in the simplest possible way.

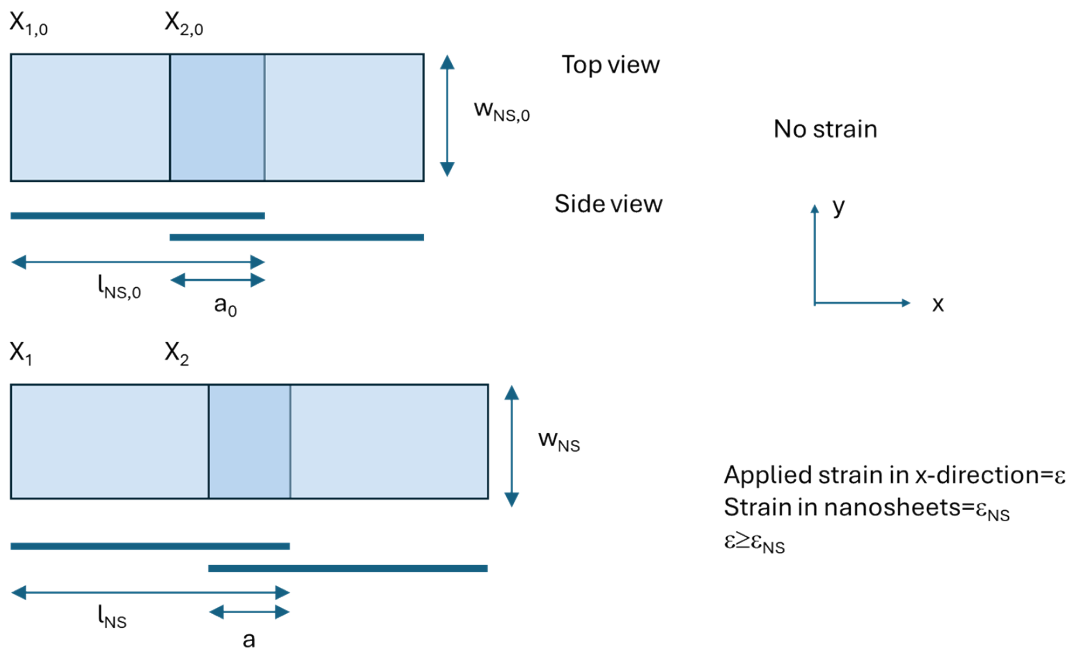

Figure S1: Nanosheet overlap schematic, showing top view and side view of two rectangular overlapping nanosheets. Upper: no strain. Lower: strain, ε, is applied in the x direction. However, we assume not all of the applied strain is transferred to the nanosheets such that the strain in the nanosheets is ε_{NS}.

Consider two rectangular nanosheets (zero-strain dimensions $l_{NS,0}$, $w_{NS,0}$) partially overlapping as shown in figure S1 (assuming we have rectangular nanosheets). The upper portion shows the zero-strain situation, where the overlapping area is the junction. Before applying strain, the junction area is $A_{J,0} = a_0 w_{NS,0}$, where a_0 is the length of the partial overlap. The left-hand edge of the nanosheets are at positions $X_{1,0}$ and $X_{2,0}$. Then the distance between edges is $(X_{2,0} - X_{1,0}) = \Delta X_0 = l_{NS,0} - a_0$.

The lower portion shows the situation after an external uniaxial strain, ε, has been applied. However, we assume that not all of the applied strain is transferred to the nanosheets such that the strain in the nanosheets is ε_{NS}.

Under strain, all dimensions change such that $(X_2 - X_1) = \Delta X = l_{NS} - a$. When strain is applied to the system, the left-hand edges of the nanosheets will become further apart: $\Delta X = (\varepsilon + 1)(\Delta X)_0$ where the subscript "0" denotes the unstrained case. Then: $\Delta X_0(\varepsilon + 1) = l_{NS} - a$ where l_{NS} and a are the values in the presence of strain. Then $(l_{NS,0} - a_0)(\varepsilon + 1) = l_{NS} - a$.

Because the strain in the nanosheets is assumed to be ε_{NS}, different to the applied strain, we have $l_{NS} = (\varepsilon_{NS} + 1) l_{NS,0}$. Then:

$$(l_{NS,0} - a_0)(\varepsilon + 1) = (\varepsilon_{NS} + 1) l_{NS,0} - a$$

Multiplying across by the width of the nanosheet under strain, w_{NS}:

$$w_{NS}(l_{NS,0} - a_0)(\varepsilon + 1) = w_{NS}(\varepsilon_{NS} + 1) l_{NS,0} - w_{NS} a$$

We assume the width of the nanosheet is reduced under strain via the Poisson effect: $w_{NS} = w_{NS,0}(1 - \nu_{NS}\varepsilon_{NS})$, where ν_{NS} is the in plane Poisson ratio of the nanosheet.

$$(w_{NS,0} l_{NS,0} - w_{NS,0} a_0)(\varepsilon + 1)(1 - \nu_{NS}\varepsilon_{NS}) = w_{NS,0} l_{NS,0}(\varepsilon_{NS} + 1)(1 - \nu_{NS}\varepsilon_{NS}) - w_{NS} a$$

But remembering that $A_J = a w_{NS}$ and writing the initial nanosheet area as $A_{NS,0} = w_{NS,0} l_{NS,0}$

$$(A_{NS,0} - A_{J,0})(\varepsilon + 1)(1 - \nu_{NS}\varepsilon_{NS}) = A_{NS,0}(\varepsilon_{NS} + 1)(1 - \nu_{NS}\varepsilon_{NS}) - A_J$$

Where $A_{J,0}$ is the unstrained junction resistance.

Rearranging

$$A_J = A_{NS,0}(1-\nu_{NS}\varepsilon_{NS})(\varepsilon_{NS}-\varepsilon)+(A_{J,0})(\varepsilon+1)(1-\nu_{NS}\varepsilon_{NS})$$

$$A_J = A_{J,0}\left[1+\frac{A_{NS,0}}{A_{J,0}}\varepsilon_{NS}+\left(1-\frac{A_{NS,0}}{A_{J,0}}\right)\varepsilon\right](1-\nu_{NS}\varepsilon_{NS})$$

The junction area is some fraction of the nanosheet area: $f_{J,0} = A_{J,0} / A_{NS,0}$. Then

$$A_J = A_{J,0}\left[1+\frac{1}{f_{J,0}}\varepsilon_{NS}+\left(1-\frac{1}{f_{J,0}}\right)\varepsilon\right](1-\nu_{NS}\varepsilon_{NS})$$

We note that there are two possible extremes:

When no strain is transferred to the nanosheets: $\varepsilon_{NS}=0$, then $A_J = A_{J,0}\left[1+\left(1-\frac{1}{f_{J,0}}\right)\varepsilon\right]$

When maximal strain is transferred to the nanosheets: $\varepsilon_{NS}=\varepsilon$, then $A_J = A_{J,0}\left[1+\varepsilon\right](1-\nu_{NS}\varepsilon)$

It is convenient to introduce a strain transfer coefficient, k : $\varepsilon_{NS}=k\varepsilon$, which much lie between 0 and 1. Then

$$A_J = A_{J,0}\left[1+\left(1-\frac{1-k}{f_{J,0}}\right)\varepsilon\right](1-\nu_{NS}k\varepsilon) \qquad \text{(S5)}$$

We can use the junction area to calculate the junction resistance by assuming: $R_J=\frac{(RA)_J}{A_J}$.

Then, in general, in the presence of strain, the junction resistance is

$$R_J \approx \frac{(RA)_J}{A_{J,0}\left[1+\left(1-\frac{1-k}{f_{J,0}}\right)\varepsilon\right](1-\nu_{NS}k\varepsilon)}$$

Using an approximation appropriate for small strain [$(1+x)^{-1} \approx 1-x$]:

$$R_J \approx \frac{(RA)_J}{A_{J,0}}\left[1-\left(1-\frac{1-k}{f_{J,0}}\right)\varepsilon\right](1+\nu_{NS}k\varepsilon) \qquad \text{(S6)}$$

This equation describes the effect of strain-induced changes in junction area on the junction resistance. However, it is likely that there is another contribution to the effects of strain on R_J.

When the nanosheets themselves are stretched, their band-structure changes, leading to changes in nanosheet resistivity and so nanosheet resistance. These changes are quantified via the nanosheet gauge factor. However, these strain-induced band-structure changes can also affect the inter-nanosheet charge transfer, i.e. by modifying the hopping rate. Indeed, it has been shown empirically that the junction resistance scales roughly linearly with the nanosheet resistivity.[4]

Here we attempt to incorporate the band-structure-related effects of strain on junction resistance into our model. We first make the assumption that strain affects both nanosheet and junction resistance in the same way, e.g. if strain increases the number of states at the Fermi energy, this will impact both the nanosheet resistivity and the inter-sheet hopping rate (i.e. the junction resistance). Thus, we make the assumption that $(RA)_J = \alpha R_{NS}$, where α is an unknown parameter with units m^2.

Then

$$R_J \approx \frac{\alpha R_{NS}}{A_{J,0}}\left[1-\left(1-\frac{1-k}{f_{J,0}}\right)\varepsilon\right](1+\nu_{NS}k\varepsilon)$$

Expanding and ignoring the ε^2 term (valid at low strain, reasonable as gauge factor is usually defined at low strain):

$$R_J \approx \frac{\alpha R_{NS}}{A_{J,0}}\left[1-\left(1-\frac{1-k}{f_{J,0}}-\nu_{NS}k\right)\varepsilon\right]$$

Differentiating yields:

$$\frac{dR_J}{d\varepsilon}\approx\frac{\alpha}{A_{J,0}}\left\{\frac{dR_{NS}}{d\varepsilon}-\left(1-\frac{1-k}{f_{J,0}}-\nu_{NS}k\right)\left(\frac{dR_{NS}}{d\varepsilon}\varepsilon+R_{NS}\right)\right\}$$

We then note the nanosheet gauge factor is defined by $(R_{NS}-R_{NS,0})/R_{NS,0}=G_{NS}\varepsilon_{NS}$, where $\varepsilon_{NS}=k\varepsilon$, this means $R_{NS}=G_{NS}R_{NS,0}\varepsilon k+R_{NS,0}$ $(dR_{NS}/d\varepsilon)=G_{NS}kR_{NS,0}$. We can use these in the equation directly above to obtain:

$$\frac{dR_J}{d\varepsilon}\approx\frac{\alpha}{A_{J,0}}\left\{G_{NS}kR_{NS,0}-\left(1-\frac{1-k}{f_{J,0}}-\nu_{NS}k\right)\left(2G_{NS}kR_{NS,0}\varepsilon+R_{NS,0}\right)\right\}$$

Using equation S3, using the relations above, simplifying and taking the limit of low strain:

$$G_{Net}=(1+\nu_y+\nu_z)+\frac{G_{NS}kR_{NS,0}+\frac{\alpha R_{NS,0}}{A_{J,0}}\left\{G_{NS}k-\left(1-\frac{1-k}{f_{J,0}}-\nu_{NS}k\right)\right\}}{R_{NS,0}+R_{J.0}}$$

Noting the definition of α and remembering that $\frac{(RA)_J}{A_{J,0}}=R_{J,0}$ (equation S4) yields:

$$G_{Net}=(1+\nu_y+\nu_z)+\frac{R_{NS,0}kG_{NS}+R_{J.0}\left\{k(G_{NS}+\nu_{NS})+\left(\frac{1-f_{J,0}-k}{f_{J,0}}\right)\right\}}{R_{NS,0}+R_{J.0}}$$

We can now apply the network conductivity model, equation 2, (written for zero strain) $\rho_{Net,0}\approx 2t_{NS}\left[R_{NS,0}+R_{J,0}\right]$ and the equivalent equation for individual nanosheets $\rho_{NS,0}\approx 2t_{NS}R_{NS,0}$ and use these to eliminate the resistances from the equation above. N.B. in the latter equation, the assumption is that the current flows on average through half the nanosheet length while the effective nanosheet cross sectional area is its length times thickness (modelling the nanosheets as square). Then the nanosheet length is removed from the resistivity equation by cancelation. Utilising the equations above and simplifying yields:

$$G_{Net}=(1+\nu_y+\nu_z)+\frac{\rho_{NS,0}kG_{NS}+(\rho_{Net,0}-\rho_{NS,0})\left\{k(G_{NS}+\nu_{NS})+\left(\frac{1-f_{J,0}-k}{f_{J,0}}\right)\right\}}{\rho_{Net,0}}$$

Simplifying

$$G_{Net} = (1+\nu_y+\nu_z) + \frac{\rho_{NS,0}}{\rho_{Net,0}} kG_{NS} + \left(1-\frac{\rho_{NS,0}}{\rho_{Net,0}}\right)\left\{k(G_{NS}+\nu_{NS}) + \left(\frac{1-f_{J,0}-k}{f_{J,0}}\right)\right\} \quad \text{(S7)}$$

This equation has two well defined limits:

1. When no strain is transferred to the nanosheets such that they slide past each other under strain. Then $k=0$ and

$$G_{Net} = (1+\nu_y+\nu_z) + \left(1-\frac{\rho_{NS,0}}{\rho_{Net,0}}\right)\left(\frac{1-f_{J,0}}{f_{J,0}}\right)$$

2. When strain is fully transferred to the nanosheets such that $k=1$

$$G_{Net} = 2\nu_y + \nu_z + G_{NS} + \left(\frac{\rho_{NS,0}}{\rho_{Net,0}}\right)(1-\nu_{NS})$$

We note that in the first case, the network gauge factors is always positive while in the second case, it can be negative is G_{NS} is negative enough to overcome the other terms.

We can use equation S7 to plot G_{Net} versus k so long as the other parameters are known. We can approximate $\nu_y = 0.5$ (that of the polymer substrate) and $\nu_z = 0$ (consistent with networks).[5-11] Carey et al. showed that for networks of electrochemically exfoliated $PtSe_2$ nanosheets,[12] the intrinsic mobility of the nanosheets was μ_{NS} = 18 cm^2/Vs, while the mobility of their overall network was μ_{Net} = 1.1 cm^2/Vs. This discrepancy comes from the fact that $R_{NS} << R_J$ in these networks. Assuming the carrier density to be the same in both nanosheets and network allows us to write: $\rho_{NS,0}/\rho_{Net,0} = \mu_{Net}/\mu_{NS} \sim 0.06$ for networks of electrochemically exfoliated $PtSe_2$ nanosheets. In addition, a value of G_{NS} = -85, was measured by Wagner et al. for thermally grown $PtSe_2$ films.[13] In a 2D system such as a nanosheet, area conservation implies $\nu_{NS} = 1$, while we assume $f_{J,0} \approx 0.4$ as reported previously.[14]

Using these numerical values and equation S7, G_{Net} is plotted versus k in Figure S2. This allows us to show graphically that a network gauge factor of -4.45 is consistent with a stress transfer factor of $k = 0.085$.

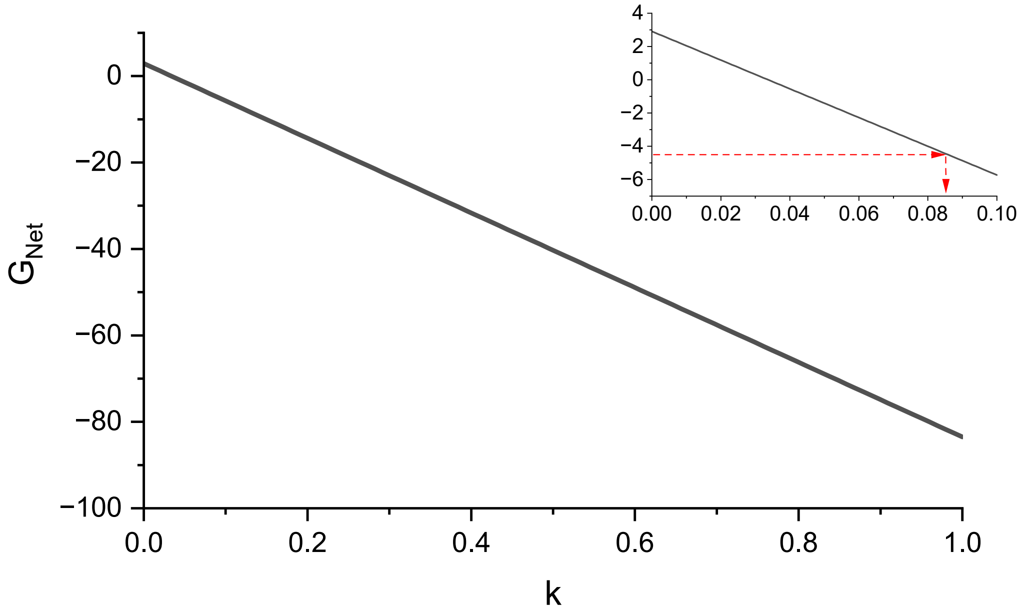

Figure S2: **Plot of** G_{Net} versus k using equation S7 and the parameters given above. This allows us to show graphically that a network gauge factor of -4.45 is consistent with a stress transfer factor of $k = 0.085$.

Measurement Set-up and Cycling the Sensor

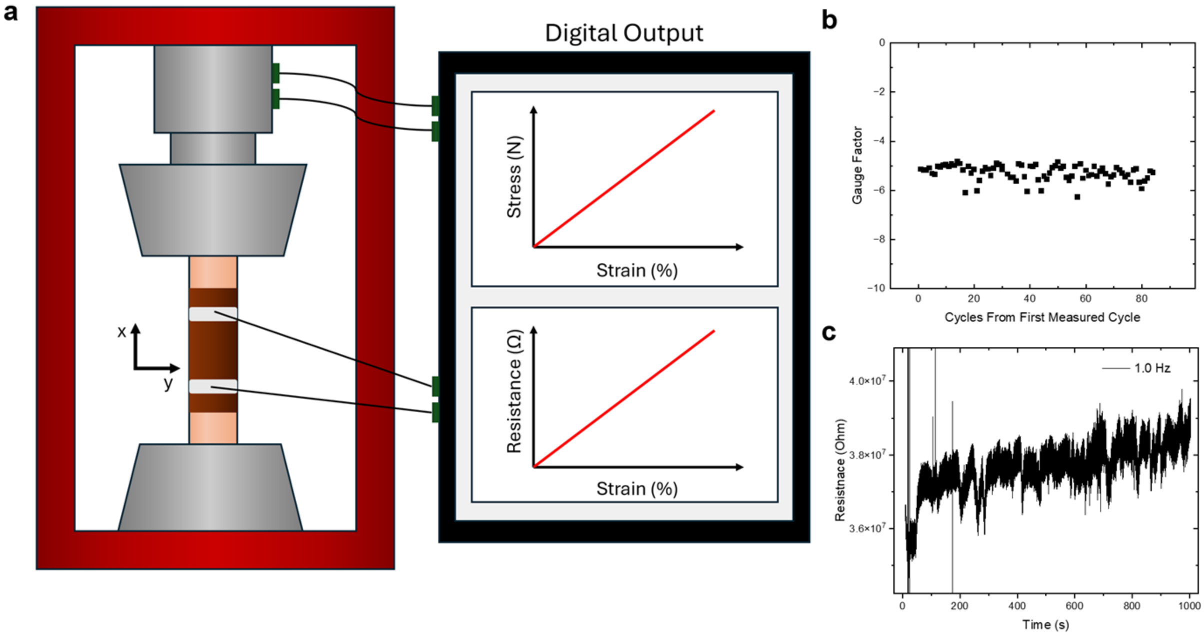

Figure S3: **a** Schematic of the tensile testing setup used in this experiment **b** The calculated gauge factor in our sensor as a function of the number of measurement cycles since the first measurement. **c** PtSe2 sensor resistance as a function of time for 1000 strain cycles. There is resistance drift of about 5% which we attribute to we attribute to temperature fluctuations, contact bonding issues or environmental doping.[15]

References

(1) Window, A. L. *Strain Gauge Technology*; Springer, 1992.
(2) Fiorillo, A. S.; Critello, C. D.; Pullano, S. A. Theory, technology and applications of piezoresistive sensors: A review. *Sensors and Actuators A: Physical* **2018**, *281*, 156-175. DOI: 10.1016/j.sna.2018.07.006.
(3) Fiorillo, A. S.; Critello, C. D.; Pullano, S. A. Theory, technology and applications of piezoresistive sensors: A review. *Sens. Actuators, A* **2018**, *281*, 156-175. DOI: https://doi.org/10.1016/j.sna.2018.07.006.
(4) Gabbett, C.; Kelly, A. G.; Coleman, E.; Doolan, L.; Carey, T.; Synnatschke, K.; Liu, S.; Dawson, A.; O'Suilleabhain, D.; Munuera, J.; et al. Understanding how junction resistances impact the conduction mechanism in nano-networks. *Nat Commun* **2024**, *15* (1), 4517. DOI: 10.1038/s41467-024-48614-5 From NLM PubMed-not-MEDLINE.
(5) Kim, K. H.; Oh, Y.; Islam, M. F. Mechanical and Thermal Management Characteristics of Ultrahigh Surface Area Single-Walled Carbon Nanotube Aerogels. *Advanced Functional Materials* **2012**, *23* (3), 377-383. DOI: 10.1002/adfm.201201055.
(6) Mecklenburg, M.; Schuchardt, A.; Mishra, Y. K.; Kaps, S.; Adelung, R.; Lotnyk, A.; Kienle, L.; Schulte, K. Aerographite: ultra lightweight, flexible nanowall, carbon microtube material with outstanding mechanical performance. *Adv Mater* **2012**, *24* (26), 3486-3490. DOI: 10.1002/adma.201200491.
(7) Wu, Y.; Yi, N.; Huang, L.; Zhang, T.; Fang, S.; Chang, H.; Li, N.; Oh, J.; Lee, J. A.; Kozlov, M.; et al. Three-dimensionally bonded spongy graphene material with super compressive elasticity and near-zero Poisson's ratio. *Nat Commun* **2015**, *6* (1), 6141. DOI: 10.1038/ncomms7141
(8) Woo, S.; Park, H. C.; Son, Y.-W. Poisson's ratio in layered two-dimensional crystals. *Physical Review B* **2016**, *93* (7), 075420. DOI: 10.1103/PhysRevB.93.075420.
(9) Zhan, H.; Guo, D.; Xie, G. Two-dimensional layered materials: from mechanical and coupling properties towards applications in electronics. *Nanoscale* **2019**, *11* (28), 13181-13212. DOI: 10.1039/c9nr03611c
(10) Fan, Y.; Xiang, Y.; Shen, H. S. Temperature-Dependent Mechanical Properties of Graphene/Cu Nanocomposites with In-Plane Negative Poisson's Ratios. *Research (Wash D C)* **2020**, *2020*, 5618021. DOI: 10.34133/2020/5618021
(11) Rawson, S. D.; Bayram, V.; McDonald, S. A.; Yang, P.; Courtois, L.; Guo, Y.; Xu, J.; Burnett, T. L.; Barg, S.; Withers, P. J. Tailoring the Microstructure of Lamellar Ti(3)C(2)T(x) MXene Aerogel by Compressive Straining. *ACS Nano* **2022**, *16* (2), 1896-1908. DOI: 10.1021/acsnano.1c04538
(12) Carey, T.; Synnatschke, K.; Ghosh, G.; Anzi, L.; Caffrey, E.; Coleman, E.; Lin, C.; Dawson, A.; Liu, S.; Wells, R.; et al. A Portfolio of Electrochemically Exfoliated Two-Dimensional Materials: From Crystals and Simulations to Electronic Inks and Circuits. *10.21203/rs.3.rs-5319871/v1* **2024**.
(13) Wagner, S.; Yim, C.; McEvoy, N.; Kataria, S.; Yokaribas, V.; Kuc, A.; Pindl, S.; Fritzen, C. P.; Heine, T.; Duesberg, G. S.; Lemme, M. C. Highly Sensitive Electromechanical Piezoresistive Pressure Sensors Based on Large-Area Layered PtSe(2) Films. *Nano Lett.* **2018**, *18* (6), 3738-3745. DOI: 10.1021/acs.nanolett.8b00928
(14) Gabbett, C.; Kelly, A. G.; Coleman, E.; Doolan, L.; Carey, T.; Synnatschke, K.; Liu, S.; Dawson, A.; O'Suilleabhain, D.; Munuera, J.; et al. Understanding how junction resistances impact the conduction mechanism in nano-networks. *Nature Communications* **2024**, *15* (1), 4517. DOI: 10.1038/s41467-024-48614-5.
(15) Yuan, T.; Yin, R.; Li, C.; Xing, J.; Jiang, D.; Fan, Z.; Pan, L. Synergistic Structural Construction of Strain Sensors with Low Baseline Drift and High Sensitivity for Continuous Dynamic Monitoring. *Nano Letters* **2025**, *25* (12), 4969-4978. DOI: 10.1021/acs.nanolett.5c00327.
